# Administering the maternal bovine appeasing substance improves overall productivity and health in high-risk cattle during a 60-d feedlot receiving period

**DOI:** 10.1093/jas/skae221

**Published:** 2024-08-03

**Authors:** Autumn T Pickett, Reinaldo F Cooke, Izadora S de Souza, Shea J Mackey

**Affiliations:** Department of Animal Science, Texas A&M University, College Station, TX 77843, USA; Department of Animal Science, Texas A&M University, College Station, TX 77843, USA; Department of Animal Science, Texas A&M University, College Station, TX 77843, USA; Department of Animal Science, Texas A&M University, College Station, TX 77843, USA

**Keywords:** maternal appeasing substance, feedlot receiving, health, performance, stress

## Abstract

This experiment evaluated health, physiological, and performance responses of high-risk cattle administered the maternal bovine appeasing substance (**mBAS**) during feedlot receiving. Angus-influenced, newly weaned male calves (*n* = 120) were purchased from an auction facility. Calves arrived at the research feedyard on day −1 and body weight (**BW**) was recorded upon arrival (199 ± 1 kg). Calves were ranked by arrival BW and received 1 of 2 treatments prior to initial processing (day 0): (1) 10 mL of an mBAS (Ferappease; FERA Diagnostics and Biologicals; College Station, TX) or (2) 10 mL of mineral oil (**CON**; placebo). Treatments were applied topically to the nuchal skin area (5 mL) and above the muzzle (5 mL). Calves were vaccinated against *Clostridium* and respiratory pathogens, dewormed, implanted, band-castrated, and received metaphylaxis at initial processing, and then distributed into 10 drylot pens according to arrival BW and treatment (*n* = 12 calves/pen, 5 pens/treatment). Calves were reapplied treatments (mBAS or CON) concurrently with booster vaccination on d 14. Feed intake and incidence of bovine respiratory disease (**BRD**) were recorded daily. Blood and hair samples from the tail-switch were collected on days 0, 14, 28, 42, and 60 for analysis of physiological variables. Calves were sampled for nasal microbiota analysis via swab on days 0, 14, and 28. Final shrunk BW was recorded on day 61 after 16 h of feed and water restriction. Calf BW gain and final BW did not differ between treatments (*P* ≥ 0.40). Incidence of BRD was similar (*P* = 0.99) between mBAS and CON (56.7% for both treatments; SEM = 6.45). A greater (*P* = 0.04) proportion of mBAS calves diagnosed with BRD required a single antibiotic treatment to regain health (70.6 vs. 47.0%; SEM = 8.32), and mortality was greater (*P* = 0.03) in CON calves diagnosed with BRD (17.6 vs. 2.94%; SEM = 5.133). Relative abundance of *Mycoplasma* in nasal microbiota was reduced (*P* = 0.04) in mBAS calves compared with CON (34.7 vs. 27.4%; SEM = 2.35). Cortisol concentration in hair from the tail-switch was less (*P* = 0.01) on day 28 for mBAS calves compared with CON. Administering mBAS improved (*P *= 0.04) total pen-based liveweight change during the experiment (498 vs. 309 kg/pen; SEM = 65.2) and final pen-based total liveweight (2,676 vs. 2,484 kg/pen; SEM = 65.4). Administration of mBAS to high-risk cattle decreased physiological stress markers, reduced mortality, and increased pen-based productivity during a 60-d receiving period.

## Teaser

Beef cattle are exposed to several stressors during the initial days in the feedlot, which impair their immunity and lead to bovine respiratory disease. This study evaluated the maternal bovine appeasing substance, an analogue of the bovine appeasing pheromone, as a strategy to alleviate such stressors and improve health and productivity of feedlot cattle.

## Introduction

The stress and health challenges that beef cattle experience during feedlot receiving are well known (Cooke, 2017), which lead to immunosuppression and elevated incidence of bovine respiratory disease (**BRD**; Duff and Galyean, 2007). In a recent review, Galyean et al. (2022) described novel management developments to mitigate stress or alleviate its consequences to immunity of receiving cattle, including administering the maternal bovine appeasing substance (**mBAS**; Colombo et al., 2020). The mBAS includes a mixture of fatty acids that replicate the composition of the original bovine appeasing pheromone, which promotes cow-calf bonding via recognition of maternal odors by the offspring (Cappellozza and Cooke, 2022). Administration of mBAS has been used to mitigate the physiological consequences of stress in cattle (Cappellozza et al., 2020; Colombo et al. 2020; Schubach et al., 2020).

Colombo et al. (2020) applied mBAS to high-risk steers at feedlot arrival, and reported an improvement in health and performance responses during a 45-d receiving period. These authors indicated the need for additional research to investigate mBAS to feedlot receiving cattle, including multiple mBAS administrations to cattle subjected to other stressors and management typical of commercial receiving yards. Examples of stressors include castration and metaphylaxis of lightweight cattle upon arrival, which are relevant practices to the US feedlot industry (NAHMS, 2013a; NAHMS. 2013b). Administration of mBAS to male calves at castration improved body weight (**BW**) gain during the following 30 d compared with cohorts that received a placebo treatment (Cappellozza and Cooke, 2022). Colombo et al. (2020) also observed that mBAS administration at arrival improved the response of steers diagnosed with BRD to the first therapeutic antimicrobial treatment. Therefore, we hypothesized that mBAS will improve productivity and immunity of high-risk cattle that are castrated and receiving metaphylaxis during initial processing. To test this hypothesis, the current experiment evaluated growth, physiological, and health responses of high-risk lightweight cattle administered mBAS during a 60-d feedlot receiving period.

## Materials and Methods

The current experiment was conducted at the Texas A&M—Beef Cattle Systems (College Station, TX) from September to November 2023. All animals were cared for in accordance with acceptable practices and experimental protocols reviewed and approved by the Texas A&M AgriLife Research, Agriculture Animal Care and Use Committee (#2021-0308).

### Animals and Treatments

Angus-influenced (*n* = 120) recently weaned, non-castrated male calves were purchased from a commercial auction facility in southwestern Tennessee and used in this experiment. No information regarding source nor previous health and management history were available. On the day of purchase (day −2; 1800 hours), calves were loaded into 2 commercial livestock trailers (Legend 50’ cattle liner; Barrett LLC., Purcell, OK) and transported for 700 km (12 h) to the experimental facility (College Station, TX). On day −1 of the experiment (0600 hours), calves were unloaded, immediately weighed [arrival BW (average ± SEM) = 199 ± 1.2 kg], and then rested as a single group for 24 h with ad libitum access to bermudagrass hay (*Cynodon dactylon*), water, and a commercial mineral + vitamin mix (Table 1). On day 0, calves were ranked according to arrival BW and assigned to receive mBAS (Ferappease; FERA Diagnostics and Biologicals; College Station, TX; *n* = 60) or placebo (mineral oil; **CON**, *n* = 60) in a manner that treatments groups had equivalent arrival BW. The active ingredient of mBAS is based on a proprietary mixture of fatty acids including palmitic, oleic, and linoleic acids, added at 10% of the excipient and estimated to remain in treated animals for 15 d (Schubach et al., 2020). Calves were segregated by treatment (2 groups) and immediately processed again for treatment administration, with CON calves processed first to avoid cross-contamination during treatment application (Colombo et al., 2020). Treatments (10 mL) were applied topically to the nuchal skin area (5 mL) and above the muzzle (5 mL) using the applicator provided by the manufacturer (FERA Diagnostics and Biologicals).

**Table 1.** Composition and nutritional profile of the total mixed ration offered for ad libitum consumption to calves during the experiment.

Item	Value
Composition, dry matter basis	
Cracked corn, %	37.1
Dried distillers’ grains, %	20.9
Alfalfa hay, %	30.7
Liquid molasses, %	8.52
Mineral mix^1^, %	2.82
Nutritional profile,^2^ dry matter basis	
Net energy for maintenance, Mcal/kg	1.74
Net energy for gain, Mcal/kg	1.12
Total digestible nutrients, %	73.0
Neutral detergent fibre, %	25.0
Crude protein, %	16.5

^1^Containing 21% Ca, 0.01% P, 21% NaCl, 0.20% K, 0.10% Mg, 0.045% Cu, 0.001% Se, 0.280% Zn, 220,000 IU/kg of vitamin A, 19,800 IU/kg of vitamin D3, and 3,500 IU/kg of vitamin E (Anipro Xtraperformance Feeds, College Station, TX). Also contained sodium monensin (Rumensin; Elanco Animal Health, Greenfield, IN) at 1,320 g/ton (37.2 g/ton of total mixed ration, dry matter basis).

^2^Based on wet chemistry procedures by a commercial laboratory (Dairy One Forage Laboratory, Ithaca, NY). Net energy for maintenance and gain were calculated using the equations for energy value of feeds described by NASEM (2016).

Immediately after treatment application on day 0, calves received initial processing that included administration of vaccines against *Clostridium chauvoei*, *C. septicum*, *C. novyi* type B, *C. haemolyticum*, *C. tetani*, and *C. perfringens* types C and D (Covexin 8; Merck Animal Health, Madison, NJ), *Mannheimia haemolytica*, bovine respiratory syncytial virus (**BRSV**), bovine herpesvirus-1 (**BoHV-1**), bovine viral diarrhea virus (**BVD)** 1 and 2, and parainfluenza-3 virus (**PI3**; Vista Once SQ; Merck Animal Health), anthelmintic (Safe-Guard, Merck Animal Health), growth-promoting implant (Synovex Choice containing 100 mg trenbolone acetate/14 mg estradiol benzoate; Zoetis, Florham Park, NJ), tulathromycin at 2.5 mg/kg of BW (Draxxin; Zoetis), and band-castration (Callicrate Pro-Bander; No-Bull Enterprises, LLC St. Francis, KS). Calves within treatment were ranked again by arrival BW, and allocated to 1 of 10 drylot pens (20 × 10 m; 12 calves/pen; *n* = 5/treatment) in a manner that all pens had equivalent arrival BW. Pens were arranged in 2 rows of 5 pens/row, and rows were assigned to mBAS and CON pens to preserve distance between pens from different treatments (Schubach et al., 2020). Treatment groups switched rows every 14 d to account for any potential row effects (days 14, 28, and 42).

From days 0 to 60, calves had free-choice access to water and a total-mixed ration (**TMR**; Table 1) that was offered once daily (0800 hours), in a manner to yield 10% residual orts (as-fed basis; Schubach et al., 2020). On day 14 of the experiment, calves were re-vaccinated against BRSV, BoHV-1, BVD types 1 and 2, and PI3 (Vista 5; Merck Animal Health) and received another 10 mL of the assigned treatment (5 mL in the nuchal skin area + 5 mL above the muzzle).

### Sampling

Samples of the TMR were collected weekly, pooled across weeks, and analyzed for nutrient content (Dairy One Forage Laboratory, Ithaca, NY). Calf full BW was recorded on days 0, 14, 28, 42, and 60 before the feeding of the day. Shrunk BW was recorded on day 61 after 16 h of feed and water restriction, and calf average daily gain (**ADG**) was calculated using arrival and final shrunk BW. Calf BW gain during the experiment was also calculated by modeling linear regression of full BW against sampling days. Feed intake (dry matter basis) was evaluated daily from each pen by collecting and weighing offered and non-consumed feed. Samples of offered and non-consumed feed were dried for 96 h at 50 °C in forced-air ovens for dry matter calculation. Feed intake of each pen was divided by the number of calves within each pen, and expressed as kg per calf/day. Gain to feed (**G:F**) ratio was calculated using total BW gain (final shrunk BW—arrival BW) and total feed intake of each pen during the experiment.

Calves were observed daily for BRD signs according to a 4-point scoring system based on depression, appetite, respiration, and temperature (DART; Sousa et al., 2019) beginning on day 7 to allow a 7-d post-metaphylactic interval (Coppin et al., 2022). Beginning on day 7, calves diagnosed with BRD signs received gamithromycin (Zactran; Merial, Duluth, GA) at 1 mL/25 kg of BW subcutaneously followed by a 7-d moratorium. Calves diagnosed with BRD signs after the first therapeutic antimicrobial treatment were administered florfenicol 300 mg/mL (Nuflor; Merck Animal Health, Madison, NJ) at 1 mL/7.6 kg of BW subcutaneously followed by a 4-d moratorium. Calves diagnosed with BRD signs after the second therapeutic antimicrobial treatment were administered ceftiofur crystalline free acid 200 mg/mL (Excede; Zoetis) at 1 mL/30.3 kg of BW followed by a 5-d moratorium. Calves diagnosed with BRD signs after the third therapeutic antimicrobial received oxytetracycline (Bio-Mycin 200; Boehringer Ingelheim, Ridgefield, CT) at 1 mL/10 kg of BW and were removed from the experiment. Mortality was observed daily, whereas calves deceased after removal from the experiment were not included into the mortality calculation (Colombo et al., 2021).

Total liveweight/pen was calculated by summing the BW of calves within each pen at arrival (day 0) and at the end of the experiment (shrunk BW; day 61). Hence, final shrunk BW of calves excluded from the experiment (mortality + removals) was considered zero (Colombo et al., 2021). A pen-basis economical evaluation was performed according to arrival and final total liveweight of each pen ($6.27/kg for arrival and $5.94/kg for final liveweight value, respectively), total feed used by each pen ($385/metric ton), medication ($22 for first antimicrobial treatment, $35 for second antimicrobial treatment; $20 for third antimicrobial treatment, $7.50 for the fourth antimicrobial treatment). Profit of each pen was estimated as final liveweight value—(arrival liveweight value + total feed costs + total medication costs).

Prior to initial processing and treatment administration, blood samples were collected from all calves on days 0 and 14, and then on days 28, 42, and 60 of the experiment. Moreover, 20 calves that represented average arrival BW (2 calves/pen) were selected for blood collection at 2 and 4 h after initial processing on day 0. Blood was collected into commercial blood collection tubes (Vacutainer, 10 mL; Becton Dickinson, Franklin Lakes, NJ) containing no additive for serum collection. Hair samples from the tail switch from each calf were also collected on days 0 and 14 prior to treatment administration, and on days 28, 42, and 60 as previously described (Schubach et al., 2017). Only black hair was collected as recommended by Burnett et al. (2014). A nasal cavity swab was collected from each calf as described by Pickett et al. (2023) concurrently with hair sampling on days 0, 14, and 28. Briefly, a 20-cm DNA-free sterile swab (Puritan Medical Products; Guilford, ME) was aseptically introduced (15 cm) into each nostril and rotated around the sides of the nasal passage. Swabs were placed into sterile RNAse, DNAse, pyrogen-free polypropylene tubes, kept on ice and stored at − 80 °C within 24 h of collection.

### Laboratorial analyses

#### Feed samples.

All samples were analyzed by wet chemistry procedures for concentrations of crude protein (method 984.13; AOAC, 2006), acid detergent fiber (method 973.18 modified for use in an Ankom 200 fiber analyzer, Ankom Technology Corp., Fairport, NY; AOAC, 2006), and neutral detergent fiber using a-amylase and sodium sulfite (Van Soest et al., 1991; modified for use in an Ankom 200 fiber analyzer, Ankom Technology Corp.). Nutritional profile of TMR is described in Table 1. Net energy for maintenance and gain of the TMR were calculated using the equations for energy value of feeds described by NASEM (2016).

#### Serum samples.

Blood samples were placed immediately on ice, centrifuged (2,500 × *g* for 30 min; 4 °C) for serum harvest and stored at −80 °C in triplicates on the same day of collection. Serum samples collected from 2 calves/pen at the time of initial processing, and then 2 and 4 h later were analyzed for cortisol (1-E3002; Salimetrics, LLC., State College, PA) and substance P concentrations (ADI-900-018; Enzo Life Sciences, Inc., Farmingdale, NY). All serum samples collected at initial processing (day 0) and days 14, 28, 42, and 60 were analyzed for haptoglobin concentrations (Cooke and Arthington, 2013). The intra- and inter-assay CV for these analyses were ≤9.2%.

Serum samples collected at initial processing (day 0) and days 14, 28, 42, and 60 were also analyzed for antibodies against BVDV types 1 and 2 (BVDV Total Ab X3 ELISA #99-41639; IDEXX Switzerland AG, Liebefeld-Bern, Switzerland), BoHV-1 (BHV-1 Ab ELISA #99-41459; IDEXX), and PI3 (PI3 Ab ELISA #P0652-2; IDEXX) as described by Gonda et al. (2012) and Cooke et al. (2020). However, only samples from calves not diagnosed with BRD signs were analyzed for antibodies against BRD viruses to ensure that this response was associated with vaccine efficacy rather than disease (Callan, 2001; Colombo et al., 2020; Cooke et al., 2020). The intra- and inter-assay CV were for these analyses were ≤7.1%.

#### Hair and nasal swab samples.

Cortisol was extracted from hair samples as previously described (Moya et al., 2013; Schubach et al., 2017), reconstituted in 100 μL of the buffer supplied with the ELISA cortisol kit (1-E3002; Salimetrics), and stored at −80 °C. Samples were analyzed for cortisol concentrations using the ELISA kit (1-E3002; Salimetrics), with intra- and inter-assay CV ≤ 4.2%. Nasal swabs were processed for DNA extraction and 16S rRNA gene amplicon sequencing as by Pickett et al. (2023). Samples were transferred to a 96-well plate and DNA extraction was performed using Mag-Bind Universal Pathogen 96 Kit (Omega Bio-Tek, Norcross, GA). The 16S amplicons were amplified by PCR for individual metagenomic DNA samples according to Bicalho et al. (2017). The V4 hypervariable region of bacterial/archaeal 16S rRNA gene were amplified with 515F (5ʹ-GTGCCAGCMGCCGCGGTAA-3ʹ) and 806R (5ʹ-GGACTACHVGGGTWTCTAAT-3ʹ) primers using methods for the Illumina MiSeq platform (Caporaso et al., 2012). Shannon diversity index was generated for phyla and genera of each sample (Kim et al., 2017).

### Statistical analysis

Data were analyzed as a completely randomized design, using pen as the experimental unit and Satterthwaite approximation to determine the denominator degrees of freedom for tests of fixed effects. Quantitative data were analyzed using the MIXED procedure of SAS (SAS Inst. Inc., Cary, NC), whereas binary data were analyzed using the GLIMMIX procedure of SAS (SAS Inst. Inc.) with a binomial distribution and logit link function. All models included pen(treatment) and calf(pen) as random variables, but for pen-based assessments that used pen(treatment) as random variable. Model statements for BW parameters, feed efficiency, and morbidity-related results contained the effects of treatment. Model statements for feed intake, cumulative BRD incidence, serum variables, hair cortisol, and nasal microbiota responses contained the effects of treatment, time (day or hour), and the resultant interaction. Serum variables, hair cortisol, and nasal microbiota responses were analyzed using results collected prior to treatment administration on d 0 as independent covariate in each respective analysis. The specified term for all repeated statements was day or hour, with pen(treatment) as subject for pen-based assessments, and calf(pen) as subject for all other analyses. The covariance structure used was first-order autoregressive, which provided the smallest Akaike information criterion and hence the best fit for all variables analyzed. All results are reported as least square means, or covariately-adjusted least square means for serum variables, hair cortisol, and nasal microbiota responses. Significance was set at *P* ≤ 0.05 and tendencies were determined if *P* > 0.05 and ≤ 0.10. Repeated measures are reported according to main treatment effect if the treatment × day (or hour after castration) interaction was *P* > 0.10.

## Results

### Performance and health responses

Arrival BW (day 0) was similar (*P* = 0.92) between treatments as designed (Table 2). No treatment effects were detected (*P* = 0.40) for individual calf ADG during the experiment (Table 2), resulting in similar (*P *= 0.46) final shrunk BW between mBAS and CON calves (Table 2). Feed intake and G:F were also not affected (*P* ≥ 0.20) by treatments (Table 2). The aforementioned ADG and BW results are based on calves the completed the experiment.

**Table 2.** Performance parameters during a 60-d feedlot receiving period of beef calves administered the maternal bovine appeasing substance (**mBAS**; *n* = 5) or mineral oil as placebo (**CON**; *n *= 5)^1^

Item	CON	mBAS	SEM	*P*-value
Arrival body weight (day −1),^2^ kg	199.4	199.6	1.78	0.92
Final body weight (day 61),^2^ kg	257.8	253.8	3.88	0.46
Average daily gain, kg/d	0.942	0.876	0.0542	0.40
Feed intake,^3^ kg/d	4.78	4.78	0.096	0.99
Gain to feed,^4^ kg/kg	0.179	0.166	0.0068	0.20

^1^Calves were purchased (day −2) from a commercial auction facility with no previous health and management history, transported overnight, and unloaded on day −1 at the research yard. Steers were maintained as a single group for 24 h with access to fresh water and bermudagrass (*Cynodon dactylon*) hay. Prior to initial processing on day 0, calves individually received 10 mL of the mBAS (Ferappease; FERA Diagnostics and Biologicals; College Station, TX) or mineral oil (CON). Treatments were applied topically to the nuchal skin area (5 mL) and above the muzzle (5 mL) of each steer. Initial processing included administration of vaccines, anthelmintic, growth-promoting implant, tulathromycin (2.5 mg/kg of BW), and band-castration. Calves were re-vaccinated against and received another 10 mL of the assigned treatment (5 mL in the nuchal skin area + 5 mL above the muzzle) on day 14.

^2^Body weight (**BW**) was recorded immediately upon arrival (day −1), whereas final shrunk BW was recorded after a 16-h feed and water restriction.

^3^Feed intake was evaluated by recording daily offer and measuring refusals daily from each pen, which was divided by the number of calves within each pen and expressed as kilogram per calf/d.

^4^Feed efficiency was calculated using total BW gain (based on arrival and final shrunk BW), and total feed intake (kg of dry matter) of each pen during the 60-d receiving period.

**Figure 1.**
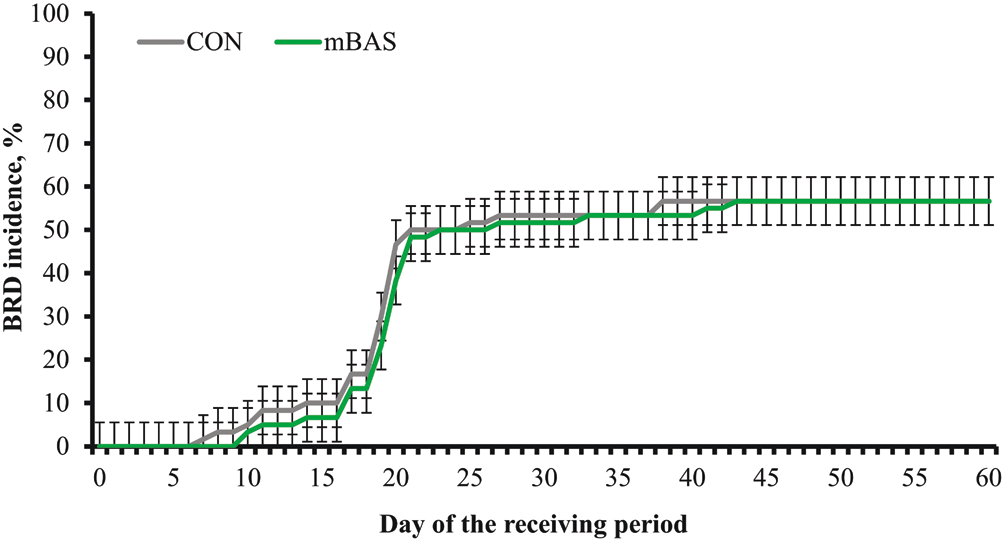
Cumulative incidence of bovine respiratory disease (BRD) signs during a 60-d feedlot receiving period of beef calves administered the maternal bovine appeasing substance (mBAS; *n* = 5) or mineral oil as placebo (CON; *n* = 5). Calves individually received 10 mL of the mBAS (Ferappease; FERA Diagnostics and Biologicals; College Station, TX) or mineral oil (CON) at initial processing (day 0) and 14 d later. Treatments were applied topically to the nuchal skin area (5 mL) and above the muzzle (5 mL) of each steer. Calves were observed daily for BRD signs according to a 4-point scoring system based on depression, appetite, respiration, and temperature (DART; Sousa et al., 2019), and received antimicrobial treatment as in Sousa et al. (2019) beginning on day 7. No treatment effect nor treatment × day interaction was detected (*P* ≥ 0.83). Values reported are least square means ± SEM.

No treatment effects were detected (*P* ≥ 0.97) for overall incidence of BRD signs (Table 3) nor timing of BRD incidence during the experiment (Figure 1). However, a greater proportion (*P* = 0.03) of mBAS calves diagnosed with BRD signs required one therapeutic antimicrobial treatment to regain health compared with CON calves (Table 3). The proportion of calves diagnosed with BRD signs that required ≥2 therapeutic antimicrobial treatments did not differ between treatments (*P* ≥ 0.59), including proportion of calves that were removed from the experiment by requiring a fourth therapeutic antimicrobial intervention (Table 3). Mortality rate was greater (*P *≤ 0.04) in CON compared with mBAS calves, when comparing calves diagnosed with BRD signs or all calves enrolled in the experiment (Table 3). The proportion of calves excluded from the experiment (mortality + removals) was also less mBAS vs. CON when comparing all calves or only those diagnosed with BRD signs (Table 3). Likewise, a greater (*P *≤ 0.05) proportion of mBAS calves completed the 60-d receiving period compared with CON (96.7 vs. 88.4% of all calves, SEM = 3.41; 94.1 vs. 79.4% of calves diagnosed with BRD, SEM = 1.52).

**Table 3.** Morbidity and mortality parameters during a 60-d feedlot receiving period of beef calves administered the maternal bovine appeasing substance (**mBAS**; *n* = 5) or mineral oil as placebo (**CON**; *n *= 5).^1,2^

Item	CON	mBAS	SEM	*P*-value
Steers treated for respiratory disease, %	56.7	56.7	6.45	0.99
One treatment required to regain health	47.0	70.6	8.32	0.03
Two treatments required to regain health	26.5	20.6	7.39	0.59
Three treatments required to regain health	5.88	2.94	3.566	0.56
Four treatments required (removals)	2.94	2.94	2.941	0.99
Overall mortality, %	10.0	1.66	3.003	0.04
Steers treated for respiratory disease	17.6	2.94	5.133	0.03
Overall mortality + removals, %	11.6	3.33	3.386	0.05
Steers treated for respiratory disease	20.6	5.88	5.760	0.04

^1^Calves were purchased (day −2) from a commercial auction facility with no previous health and management history, transported overnight, and unloaded on day −1 at the research yard. Steers were maintained as a single group for 24 h with access to fresh water and bermudagrass (*Cynodon dactylon*) hay. Prior to initial processing on day 0, calves individually received 10 mL of the mBAS (Ferappease; FERA Diagnostics and Biologicals; College Station, TX) or mineral oil (CON). Treatments were applied topically to the nuchal skin area (5 mL) and above the muzzle (5 mL) of each steer. Initial processing included administration of vaccines, anthelmintic, growth-promoting implant, tulathromycin (2.5 mg/kg of BW), and band-castration. Calves were re-vaccinated against and received another 10 mL of the assigned treatment (5 mL in the nuchal skin area + 5 mL above the muzzle) on day 14.

^2^Steers were observed daily for signs of bovine respiratory disease according to a 4-point scoring system based on depression, appetite, respiration, and temperature (DART; Sousa et al., 2019), and received antimicrobial treatment as by Sousa et al. (2019). Steers were removed from the experiment when the fourth therapeutic antimicrobial treatment was administered.

No treatment differences were detected (*P* = 0.47) for total liveweight/pen on day 0 (Table 4). Change in total liveweight/pen during the experiment and total liveweight/pen on day 61 were greater (*P* ≤ 0.04) mBAS vs. CON pens (Table 4). Total feed consumed per pen during the experiment tended to be greater (*P *= 0.09) in mBAS pens, whereas G:F was greater (*P *= 0.04) in mBAS vs. CON pens (Table 4). Pens that received mBAS had greater (*P *= 0.04) final value and profit, with a tendency (*P *= 0.09) for increased feed costs but similar (*P *= 0.53) medication costs compared with CON pens (Table 4).

**Table 4.** Productive and economical responses during a 60-d feedlot receiving period of pens containing beef calves administered the maternal bovine appeasing substance (**mBAS**; *n* = 5) or mineral oil as placebo (**CON**; *n *= 5).^1^

Item	CON	mBAS	SEM	*P*-value
Productive responses				
Arrival liveweight,^2^ kg/pen	2,175	2,178	2.6	0.47
Final liveweight (shrunk),^2^ kg/pen	2,484	2,676	65.4	0.04
Liveweight gain, kg/pen	309	498	65.2	0.04
Total feed intake,^3^ kg/pen	3,103	3,340	98.5	0.09
Gain to feed,^4^ kg/kg per pen	0.097	0.148	0.0151	0.04
Economical assessment^5^				
Initial value, $/pen	14,185	14,202	15.5	0.47
Final value, $/pen	15,350	16,534	418.4	0.04
Feed cost, $/pen	1,194	1,286	30.9	0.09
Medication cost, $/pen	265	229	30.2	0.53
Profit, $/pen	-294	816	408.8	0.04

^1^Calves were purchased (day −2) from a commercial auction facility with no previous health and management history, transported overnight, and unloaded on day −1 at the research yard. Calves were maintained as a single group for 24 h with access to fresh water and bermudagrass (*Cynodon dactylon*) hay. Prior to initial processing on day 0, calves individually received 10 mL of the mBAS (Ferappease; FERA Diagnostics and Biologicals; College Station, TX) or mineral oil (CON). Treatments were applied topically to the nuchal skin area (5 mL) and above the muzzle (5 mL) of each steer. Initial processing included administration of vaccines, anthelmintic, growth-promoting implant, tulathromycin (2.5 mg/kg of BW), and band-castration. After initial processing, calves within treatment were allocated to 1 of 10 drylot pens (12 calves/pen; *n* = 5/treatment). Calves were re-vaccinated against and received another 10 mL of the assigned treatment (5 mL in the nuchal skin area + 5 mL above the muzzle) on day 14.

^2^Sum of the body weight within each pen recorded immediately upon arrival (day −1), and final shrunk body weight recorded after a 16-h feed and water restriction on day 61 (final).

^3^Evaluated by recording daily offer and measuring refusals daily from each pen.

^4^Feed efficiency was calculated using liveweight gain and total feed intake (kg of dry matter) of each pen during the 60-d receiving period.

^5^Arrival and final value calculated using pen liveweight added a 4% shrink, and $6.27/kg for arrival and $5.94/kg for final liveweight value. Feed cost was estimated at $385/ton, and medication cost as $22 for first treatment (Zactran; Merial, Duluth, GA), $35 for second treatment (Nuflor; Merck Animal Health, Madison, NJ), $20 for third treatment (Excede; Zoetis, Florham Park, NJ), $7.50 for the fourth treatment (Bio-Mycin 200; Boehringer Ingelheim, Ridgefield, CT). The profit of each pen was estimated as final value—(arrival value + feed costs + medication costs).

### Physiological responses

**Figure 2.**
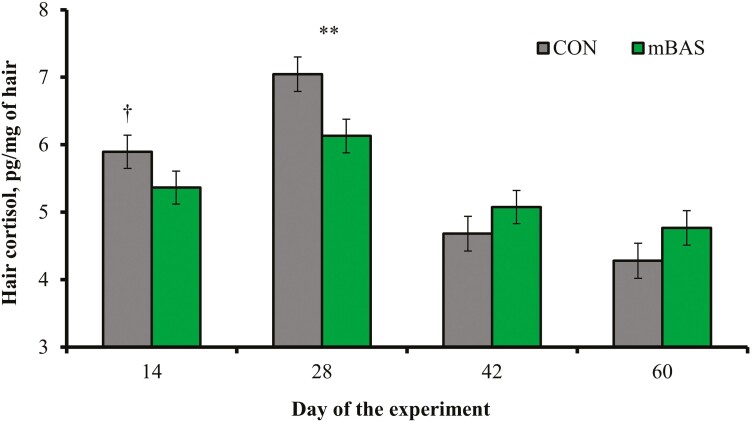
Concentrations of cortisol in hair from the tail-switch of beef calves administered the maternal bovine appeasing substance (mBAS; *n* = 5) or mineral oil as placebo (CON; *n* = 5). Calves individually received 10-mL of the mBAS (Ferappease; FERA Diagnostics and Biologicals; College Station, TX) or mineral oil (CON) at initial processing (day 0) and 14 d later. Treatments were applied topically to the nuchal skin area (5 mL) and above the muzzle (5 mL) of each steer. Hair samples were collected on days 0 and 14 prior to treatment administration, and then on days 28, 42, and 60 as in Schubach et al. (2017). Values from day 0 were used as covariate and did not differ (*P* = 0.55) between CON and mBAS calves (5.04 vs. 5.28 pg/mg of hair, respectively; SEM = 0.274); therefore, values reported are covariately adjusted least square means ± SE. A treatment × day interaction was detected (*P* < 0.01). Within day, **P = 0.01 and ^†^*P* = 0.08.

**Figure 3.**
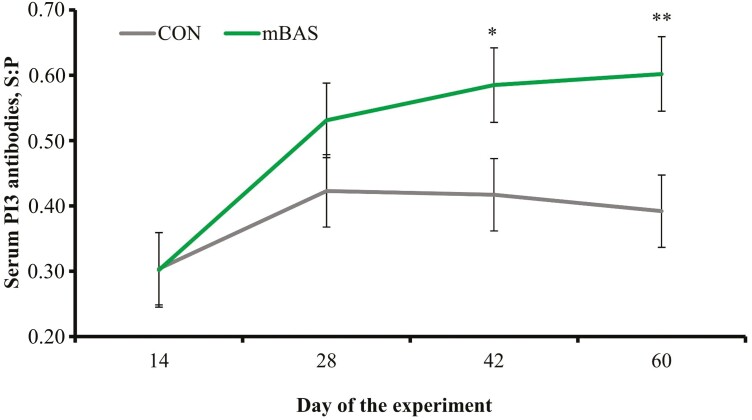
Serum concentrations of antibodies against parainfluenza-3 virus (PI3) in beef calves administered the maternal bovine appeasing substance (mBAS; *n* = 5) or mineral oil as placebo (CON; *n* = 5). Calves individually received 10-mL of mBAS (Ferappease; FERA Diagnostics and Biologicals; College Station, TX) or mineral oil (CON) at initial processing (day 0) and 14 d later. Treatments were applied topically to the nuchal skin area (5 mL) and above the muzzle (5 mL) of each calf. Calves received vaccination against respiratory pathogens on day 0 (Vista Once SQ; Merck Animal Health, Madison, NJ) and on day 14 (Vista 5; Merck Animal Health). Blood samples were collected on days 0 and 14 prior to treatment administration, and then on days 28, 42, and 60 as in Colombo et al. (2020). Results are expressed as sample:positive control ratio (S:P; Cooke et al., 2020). Values from day 0 were used as covariate and did not differ (*P* = 0.81) between CON and mBAS calves (0.279 vs. 0.301, respectively; SEM = 0.0638); therefore, values reported are covariately adjusted least square means ± SE. A treatment × day interaction was detected (*P* = 0.04). Within day, **P < 0.01 and *P = 0.03.

No treatment differences were detected (*P *= 0.97) for serum concentrations of substance P in samples collected following castration on day 0 (Table 5). However, mean concentration of serum cortisol after castration was less (*P* < 0.01) in mBAS compared with CON calves (Table 5). A treatment × day interaction was detected (*P* < 0.01) for cortisol concentration in hair from the tail-switch (Figure 2). Calves receiving mBAS had less hair cortisol concentration on day 14 (tendency; *P *= 0.08) and day 28 (*P *= 0.01) compared with CON calves. No treatment effects were detected (*P* = 0.51) for serum haptoglobin concentrations (Table 5). A treatment × day interaction was detected (*P* = 0.04) for serum concentration of antibodies against PI3 in calves not diagnosed with BRD, which was greater (*P* ≤ 0.03) in mBAS on days 42 and 60 compared with CON calves (Figure 3). No treatment effects were detected (*P* ≥ 0.81) for serum concentrations of antibodies against BVDV and BoHV-1 in calves not diagnosed with BRD (Table 5).

**Table 5.** Physiological responses of beef calves administered the maternal bovine appeasing substance (**mBAS**; *n* = 5) or mineral oil as placebo (**CON**; *n *= 5)^1^

Item	CON	mBAS	SEM	*P*-value
Responses during the day of initial processing^2^				
Serum cortisol after castration, ng/mL	36.6	25.6	2.11	< 0.01
Serum substance P after castration, pg/mL	1856	1849	138.4	0.97
Responses during the 60-d receiving period^3^				
Serum haptoglobin concentrations, mg/mL	0.693	0.771	0.0807	0.51
Serum antibodies against BVDV types 1 and 2, S:P	1.51	1.50	0.072	0.91
Serum antibodies against BoHV-1, S:P	2.29	2.26	0.104	0.81

^1^Calves were purchased (day −2) from a commercial auction facility with no previous health and management history, transported overnight, and unloaded on day −1 at the research yard. Steers were maintained as a single group for 24 h with access to fresh water and bermudagrass (*Cynodon dactylon*) hay. Prior to initial processing on day 0, calves individually received 10 mL of the mBAS (Ferappease; FERA Diagnostics and Biologicals; College Station, TX) or mineral oil (CON). Treatments were applied topically to the nuchal skin area (5 mL) and above the muzzle (5 mL) of each steer. Initial processing included administration of vaccines, anthelmintic, growth-promoting implant, tulathromycin (2.5 mg/kg of BW), and band-castration. Calves were re-vaccinated against and received another 10 mL of the assigned treatment (5 mL in the nuchal skin area + 5 mL above the muzzle) on day 14.

^2^Blood samples were collected prior to treatment administration (0 h) and at 2 and 4 h after initial processing. Results from hour 0 were used as covariate in each respective analysis, and did not differ (*P *≥ 0.20) between CON and mBAS calves (2,500 and 2,540 pg/mL for substance P, respectively, SEM = 312.2; and 28.8 and 22.4 for serum cortisol, respectively; SEM = 3.45). No treatment × hour interactions were detected (*P *≥ 0.55); therefore, values are reported as covariately adjusted least square means according to the main treatment effect.

^3^Blood samples were collected on days 0 and 14 prior to treatment administration, and on days 28, 42, and 60. Calves received vaccination against respiratory pathogens on day 0 (Vista Once SQ; Merck Animal Health, Madison, NJ) and on day 14 (Vista 5; Merck Animal Health). Results from serum antibodies against respiratory viruses are expressed as sample:positive control ratio as by Cooke et al. (2020), only within samples collected from calves not diagnosed with bovine respiratory disease during the experiment. Values from day 0 were used as covariate in each respective analysis and did not differ (*P *≥ 0.85) between CON and mBAS calves (1.28 and 1.31 mg/mL of haptoglobin, respectively, SEM = 0.116; 0.271 and 0.325 S:P for BVDV types 1 and 2, respectively, SEM = 0.1935; and 0.562 and 0.577 S:P for BoHV-1, respectively; SEM = 0.1589). No treatment × day interactions were detected (*P *≥ 0.42); therefore, values are reported as covariately adjusted least square means according to the main treatment effect.

### Nasal microbiota

The relative abundance of bacteria derived from each individual nasal swab was assigned to 29 different phyla and 864 different genera. The 5 most prevalent phylum and 10 most prevalent genus are reported in Table 6. Calves administered mBAS had decreased (*P *= 0.04) mean prevalence of the phylum Tenericutes in nasal swabs collected on days 14 and 28, with no other differences (*P* ≥ 0.34) including Shannon diversity for phylum compared with CON calves (Table 6). Similarly, mBAS administration decreased (*P* = 0.04) mean prevalence of the genera *Mycoplasma* in nasal swabs collected on days 14 and 28, with no other genera nor Shannon diversity differences (*P* ≥ 0.18) for genera compared with CON calves (Table 6).

**Table 6.** Bacterial composition (relative abundance, %) and diversity [Shannon diversity (**SD;**  Kim et al., 2017)] in the nasal cavity of beef steers administered the maternal bovine appeasing substance (**mBAS**; *n* = 5) or mineral oil as placebo (**CON**; *n *= 5)^1^

Item	CON	mBAS	SEM	*P*-value
Bacterial phyla				
Tenericutes	34.2	27.0	2.32	0.04
Proteobacteria	26.9	30.3	2.41	0.34
Firmicutes	19.2	21.2	1.66	0.42
Actinobacteria	13.0	12.9	1.37	0.95
SD index	1.25	1.28	0.028	0.45
Bacterial genera				
* Mycoplasma*	34.7	27.4	2.35	0.04
* Mannheimia*	16.8	19.3	3.00	0.57
* Corynebacterium*	5.20	5.65	0.754	0.68
* Salinicoccus*	2.41	2.69	0.376	0.61
* Cellulomonas*	1.43	1.01	0.434	0.50
* Pedobacter*	1.70	2.91	0.600	0.18
* Dietzia*	1.06	1.25	0.149	0.39
* Clostridium*	1.24	1.24	0.089	0.97
* Butyrivibrio*	1.76	2.04	0.239	0.42
* Blautia*	1.08	1.67	0.289	0.18
SD index	2.56	2.73	0.124	0.37

^1^Calves were purchased (day −2) from a commercial auction facility with no previous health and management history, transported overnight, and unloaded on day −1 at the research yard. Steers were maintained as a single group for 24 h with access to fresh water and bermudagrass (*Cynodon dactylon*) hay. Prior to initial processing on day 0, calves individually received 10 mL of the mBAS (Ferappease; FERA Diagnostics and Biologicals; College Station, TX) or mineral oil (CON). Treatments were applied topically to the nuchal skin area (5 mL) and above the muzzle (5 mL) of each steer. Initial processing included administration of vaccines, anthelmintic, growth-promoting implant, tulathromycin (2.5 mg/kg of BW), and band-castration. Calves were re-vaccinated against and received another 10 mL of the assigned treatment (5 mL in the nuchal skin area + 5 mL above the muzzle) on day 14.

^2^Steers were sampled for nasal microbiota analysis via nasal swab (Pickett et al., 2023) on days 0 and 14 prior to treatment administration, and also on day 28. Values from day 0 were used as covariate in each respective analysis and did not differ (*P *≥ 0.24) between CON and mBAS calves for relative abundance of Tenericutes (2.61% and 4.37%, respectively; SEM = 1.057) or *Mycoplasma* (2.96 and 4.53%, respectively; SEM = 1.164). No treatment × day interactions were detected (*P *≥ 0.21); therefore, values are reported as covariately adjusted least square means according to the main treatment effect.

## Discussion

The current experiment exposed newly weaned lightweight calves to the stress of transport, commingling, initial processing, band-castration, and introduction to a new environment within a 48-h period. Therefore, calves were considered high-risk to develop BRD (Wilson et al., 2017), which corroborate the substantial incidence of BRD signs observed in the current experiment. Metaphylaxis remains an important tool to mitigate BRD in high-risk cattle (NAHMS, 2013b), but does not eliminate the incidence of the disease as observed herein. Therefore, management interventions that enhance the efficacy of metaphylaxis are still warranted (Galyean et al., 2022), including mBAS that improved first-treatment success rate by Colombo et al. (2020).

### Performance and health responses

No differences were detected for ADG, feed intake, and G:F between CON and mBAS calves that completed the current 60-d receiving experiment, which is contrary to our hypothesis and previous research (Colombo et al., 2020; Schubach et al., 2020). The incidence and timing of BRD signs were also not impacted by treatments in the current experiment. Colombo et al. (2020) reported that incidence of BRD signs was not altered by mBAS administration at feedlot arrival, although BRD signs were detected earlier in steers that received mBAS. The earlier BRD detection in mBAS steers by Colombo et al. (2020) resulted in a greater proportion of mBAS steers that only required one therapeutic antimicrobial treatment (Ferran et al., 2011). Hervet et al. (2021) noted that feedlot bulls administered mBAS at arrival had increased clinical cases of BRD 8 d later, but decreased BRD occurrence 30 d after arrival compared with non-treated bulls. Number of antimicrobial treatments has been associated with BW gain and feed efficiency in feedlot cattle diagnosed with BRD (Waggoner et al., 2007; Thompson et al., 2012; Blakebrough-Hall et al., 2020). Therefore, Colombo et al. (2020) attributed the increase in ADG and G:F in steers administered mBAS to earlier BRD detection and lessened disease recurrence upon first therapeutic antimicrobial treatment.

Colombo et al. (2020) did not use metaphylaxis and noted a peak in BRD signs within 7 d after arrival. The use of metaphylaxis in the current experiment may have prevented earlier detection of BRD signs in mBAS calves (Ives and Richeson, 2015), which was mainly observed 20 d after arrival across treatments. Yet, a greater proportion of mBAS calves diagnosed with BRD signs regained health after a single therapeutic antimicrobial treatment compared with CON calves herein. Mortality of calves diagnosed with BRD signs was also reduced by mBAS administration in the current experiment, which suggests enhanced response to metaphylaxis and subsequent antimicrobial interventions (Ives and Richeson, 2015). Perhaps mBAS administration and its role in mitigating the physiological consequences of stress increased the efficiency of antimicrobial treatments (Radek, 2010), although research is warranted to validate this rationale. Colombo et al. (2020) did not observe a decrease in mortality in steers administered mBAS at arrival, which may explain the discrepancy of mBAS effects between studies on cattle growth. Cattle diagnosed with BRD experience reduced ADG and feed efficiency, even when the first therapeutic antimicrobial treatment is successful (Blakebrough-Hall et al., 2020; Pickett et al., 2023). Hence, the greater proportion of mBAS calves diagnosed with BRD that completed the current 60-d receiving experiment may have prevented a similar increase in ADG and G:F from mBAS administration as by Colombo et al. (2020).

Based on the similar calf ADG and final shrunk BW between treatments, the increase in pen-based liveweight change and final liveweight were resulted from decreased removal rate of mBAS calves in the current experiment (Colombo et al., 2021). Estimated feed costs were greater in mBAS pens as these had more calves completing the current experiment. Estimated costs with medication did not change according to treatment, despite increased efficacy of the first therapeutic antimicrobial use in mBAS calves. Accordingly, estimated final pen-based value profit were both increased by mBAS administration in the current experiment, and resulted in a return-on-investment (**ROI**) of 1,541%. This ROI was calculated based on profit differences of CON and mBAS pens ($−294 vs. $816 = $1,110) divided by $72 for mBAS costs ($3 for each individual 10 mL dose, 12 calves/pen receiving 2 doses). The pen-based assessment provides evidence of the economic benefits of mBAS to receiving yards; however, it should be interpreted and extrapolated with caution as values used for cattle, feed, and medications vary according to time and location.

### Physiological and nasal microbiota responses

Substance P is a neurotransmitter involved in the processing of noxious information to the brain, and used as indicator for nociception in cattle (Tschoner and Feist, 2022). Nociception is the detection of painful stimuli (Renthal, 2020); hence, substance P has been used as biomarker of post-castration pain in male calves (Coetzee et al., 2008). Cortisol has long been considered a biomarker of adrenocortical activity and acute stress in cattle (Carroll and Forsberg, 2007). Serum concentrations of substance P did not differ, but serum cortisol concentrations were less in calves administered mBAS compared with CON upon band-castration on day 0 of the current experiment. These results suggest that mBAS administration did not alleviate pain, but lessened the physiological stress reactions perceived from the pain of castration (Tschoner and Feist, 2022).

Cortisol concentration in hair from the tail switch is a biomarker of chronic stress (Burnett et al., 2014; Moya et al., 2015), given that cortisol is gradually accumulated in the emerging tail hair and its concentration represents long-term adrenocortical activity (Moya et al., 2013). Hence, mBAS administration also lessened chronic stress according to hair cortisol concentration on days 14 and 28 of the current experiment, which encompasses the period that the mBAS remains active (Cappellozza and Cooke, 2022). Hair cortisol concentrations in cattle can be increased by infectious diseases such as BRD (Burnett et al., 2014). Incidence of BRD signs was similar between treatments and thus had limited, if any, influence on hair cortisol results in the current experiment. Schubach et al. (2020) administered mBAS at weaning and reported that hair cortisol concentrations were less in mBAS calves on day 14 after weaning, but not on days 28 and 42 compared with calves receiving placebo. Schubach et al. (2020) also reported that mBAS administration reduced plasma haptoglobin concentration, but mainly on days 3 and 7 after weaning. The stressors associated with weaning and feedlot receiving stimulate the acute-phase response (Cooke, 2017), and heightened adrenocortical function increases circulating haptoglobin levels in cattle (Cooke and Bohnert, 2011; Cooke et al., 2012). Serum haptoglobin concentrations were not affected by mBAS administration in the current experiment nor in Colombo et al. (2020). Circulating haptoglobin in high-risk cattle often peaks within 10 d after feedlot arrival (Cooke, 2017; Sousa et al., 2019). Hervet et al. (2021) reported that feedlot bulls administered mBAS upon arrival had decreased whole blood mRNA expression of interleukin-6, a proinflammatory cytokine that triggers hepatic synthesis of haptoglobin (Carroll and Forsberg, 2007), on day 8 after arrival compared with non-treated bulls. Cattle in the current experiment and by Colombo et al. (2020) were sampled on day 14 after initial processing; therefore, after the expected haptoglobin peak which may have limited the assessment of mBAS effects on serum haptoglobin concentrations.

Vaccine efficacy is reduced when administered to highly stressed animals, which decreases protection against BRD pathogens and increase disease susceptibility (Blecha et al., 1984; Schumaher et al., 2019). Schubach et al. (2020) reported that mBAS administration at weaning increased circulating concentrations of antibodies against BRSV, BVDV, and PI3 during a 42-d preconditioning period. Kvamme et al. (2024) also observed greater serum concentrations of antibodies against BRSV and PI3 in steers vaccinated and administered mBAS at weaning. Results from the current experiment corroborate these previous studies, at least partially, as mBAS calves had greater serum concentrations of antibodies against PI3 on days 42 and 60. The benefits of mBAS to vaccine efficacy can be attributed to alleviated stress during feedlot receiving, and decreased chronic adrenocortical responses as observed in hair cortisol concentration (Cooke, 2017). The increase in serum concentrations of antibodies against PI3 in mBAS cattle, however, did not reduce the incidence of BRD signs in the current experiment nor by Colombo et al. (2020).

### Nasal microbiota

The bovine upper respiratory tract harbors a variety of microorganisms that coexist in a balanced state, whereas stress-induced immunosuppression and imbalances in this microbiota lead to BRD (Bosch et al., 2013). Tenericutes is one of the most prevalent bacterial phyla in the nasopharynx and trachea of feedlot cattle, and may comprise more than 40% of the total bacterial community (Strobel et al., 2018; Timset et al., 2018). This phylum includes the genus *Mycoplasma*, which is a major pathogen associated with BRD (Czuprynski et al., 2004; Caswell and Archambault, 2007). Calves administered mBAS in the current experiment had reduced prevalence of *Mycoplasma*, and consequently of Tenericutes, in their nasal cavity compared with CON calves. These outcomes were not sufficient to alter bacterial diversity nor the prevalence of other phyla and genera in the nasal microbiota. The nasopharyngeal microbiota is significantly altered in feedlot cattle diagnosed with BRD, including greater prevalence of *Mycoplasma* (Holman et al., 2015), although timing and incidence of BRD signs were similar between treatments in the current experiment. Besides less prevalence of *Mycoplasma* in the nasal microbiota, mBAS calves had improved response to the first therapeutic antimicrobial treatment and decreased mortality rates herein. The cause-effect relationship among these results warrant investigation; mBAS calves may have experienced reduced prevalence of *Mycoplasma* because of improved response to the antimicrobial treatment when diagnosed with BRD. Alternatively, mBAS administration may have limited prevalence of *Mycoplasma* by improving calf immunity, which increased efficacy of the first antimicrobial treatment and lessened mortality from BRD. Collectively, the nasal microbiota results from the current experiment are novel and further support the immune benefits of mBAS by alleviating the physiological consequences of stressors elicited during feedlot receiving.

### Overall conclusions

In the current experiment, mBAS administration to high-risk calves decreased physiological stress markers, improved immunocompetence parameters, and reduced mortality during a 60-d receiving period. Calves were castrated and received metaphylaxis upon arrival, which are relevant management practices in US feedyards. Calf performance and incidence of BRD signs were not affected by mBAS administration, whereas the decrease in mortality resulted in greater pen-based liveweight change and final liveweight by the end of the 60-d receiving period. Therefore, the current experiment provides additional evidence of the benefits from mBAS administration on overall productivity and health responses in receiving yards. Research is warranted to further characterize the health advantages of mBAS administration to high-risk cattle, including the increased efficacy of antimicrobial treatments in cattle diagnosed with BRD herein and by Colombo et al. (2020).

## Abbreviations

ADG: average daily gain
BoHV-1: *bovine herpesvirus-1*
BRD: bovine respiratory disease
BRSV: bovine respiratory syncytial virus
BVD: bovine viral diarrhea virus
BW: body weight
G:F: gain to feed
mBAS: maternal bovine appeasing substance
PI3: *parainfluenza-3 virus*
TMR: total mixed ration
